# Supplementary material for: Complex‐centric proteome profiling by SEC‐SWATH‐MS
Source: Mol Syst Biol. 2019 Jan 14;15(1):e8438. doi: 10.15252/msb.20188438 (PMC6346213; doi:10.15252/msb.20188438)
Supplement: Supplementary file 7 — Dataset EV6 [file MSB-15-e8438-s007.zip › feature_plots_bioplex/Q15365.pdf]

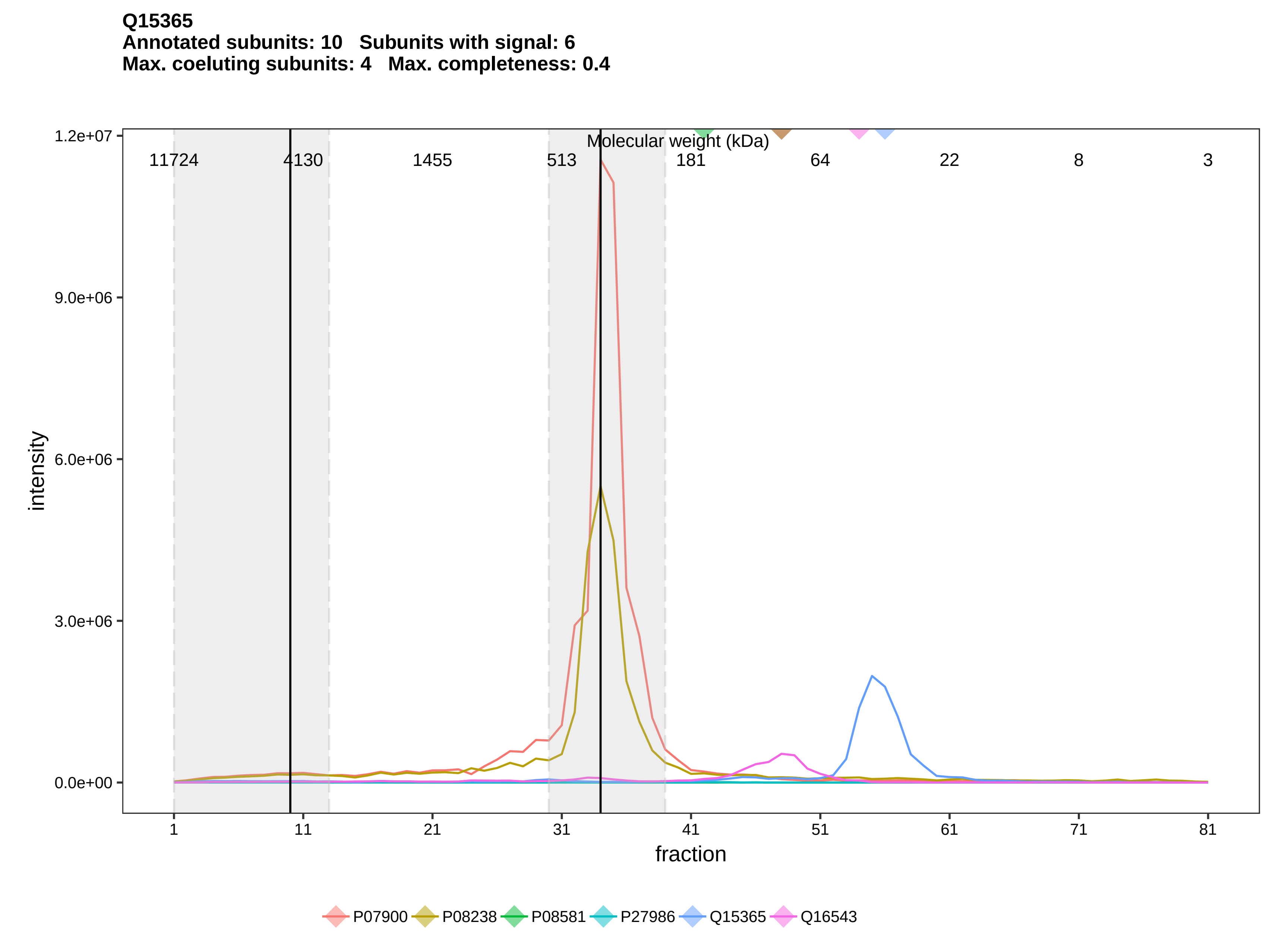
Q15365
Annotated subunits: 10 Subunits with signal: 6
Max. coeluting subunits: 4 Max. completeness: 0.4
Molecular weight (kDa)
11724
4130
1455
513
181
64
22
8
3
1.2e+07
9.0e+06
6.0e+06
3.0e+06
0.0e+00
intensity
1
11
21
31
41
51
61
71
81
fraction
P07900
P08238
P08581
P27986
Q15365
Q16543
